# Japanese encephalitis virus reprograms host pyrimidine metabolism to facilitate viral replication

**DOI:** 10.1016/j.imj.2026.100274

**Published:** 2026-07-31

**Authors:** Jun Gu, Chencheng Fan, Shengxian Xiang, Youhui Si, Bibo Zhu, Shengbo Cao, Jing Ye

**Affiliations:** a National Key Laboratory of Agricultural Microbiology, Huazhong Agricultural University, Wuhan, 430070, China; b Hubei Hongshan Laboratory, Wuhan, 430070, China; c Frontiers Science Center for Animal Breeding and Sustainable Production, College of Veterinary Medicine, Huazhong Agricultural University, Wuhan, 430070, China; d Key Laboratory of Preventive Veterinary Medicine in Hubei Province, Wuhan, 430070, China

**Keywords:** Japanese encephalitis virus, Pyrimidine metabolism, Brain, Glutamine, Aspartate, Metabolic reprogramming

## Abstract

**Background:**

Pyrimidine metabolism is crucial for the replication of viruses and the functionality of host cells. However, the cell-type-specific organization of this metabolism in the central nervous system and its dysregulation during Japanese encephalitis virus (JEV) infection remain poorly understood. Here, we aimed to characterize the cell-type-specific landscape of pyrimidine metabolism in the CNS and uncover pyrimidine metabolic reprogramming during JEV infection.

**Methods:**

Single-cell RNA sequencing data from normal and JEV-infected mouse brains were analyzed to profile the expression of pyrimidine metabolic genes across neuronal, glial, and vascular cell types. Functional validation of these metabolic pathways on JEV replication was performed in Neuro-2a cells and primary mouse brain-derived mixed neuron/glia cultures using pharmacological inhibitors, metabolite supplementation, and virological assays, including quantitative reverse transcription polymerase chain reaction, immunoblotting, and plaque assay.

**Results:**

We defined a universal core program for pyrimidine homeostasis alongside specialized, function-oriented metabolic programs across neurons, glia, and vascular cells in the normal mouse brain. JEV infection triggered cell-intrinsic reprogramming of this metabolic network, with viral replication critically depending on the *de novo* pyrimidine biosynthesis pathway in neurons. Pharmacological inhibition of the key enzyme dihydroorotate dehydrogenase significantly suppressed JEV replication. Furthermore, JEV replication created a metabolic dependency on exogenous glutamine and enhanced glutamate oxaloacetate transaminase-mediated aspartate synthesis for precursor acquisition.

**Conclusions:**

JEV rewires host brain pyrimidine metabolism in a cell-type-specific manner, revealing distinct metabolic dependencies on *de novo* pyrimidine biosynthesis, exogenous glutamine uptake, and glutamate oxaloacetate transaminase-mediated aspartate synthesis in neurons and highlighting potential targets for future antiviral strategies. By resolving these alterations at single-cell resolution, this study also provides a cell-type-resolved pyrimidine metabolic landscape of both the normal and JEV-infected brain.

## Highlights

• Our study presents the first cell-type-specific map of brain pyrimidine metabolism at single-cell resolution.
• JEV replication depends on *de novo* pyrimidine synthesis.
• JEV replication creates dependencies on glutamine uptake and GOT-mediated aspartate synthesis.

## Graphical abstract

**Figure:**
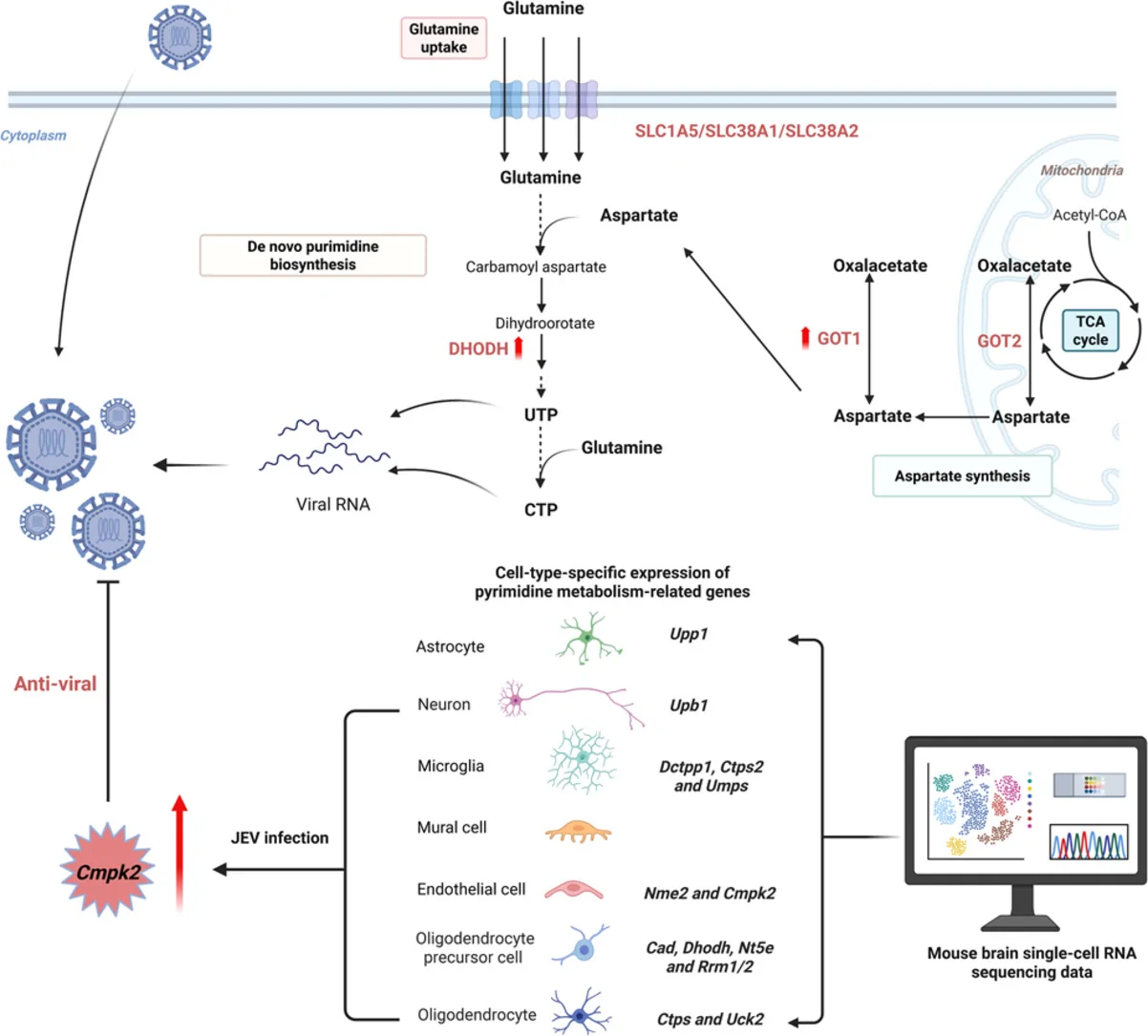

## 1 Introduction

Japanese encephalitis virus (JEV), a zoonotic mosquito-borne orthoflavivirus, is a leading cause of viral encephalitis in Asia,^1^ imposing a substantial burden on global public health^2^ and animal husbandry.^3^^,^^4^ As a positive-sense, single-stranded RNA virus with a genome of approximately 11 kilobases in length,^5^ JEV intrinsically relies on RNA molecules for its entire replication cycle in neurons. This fundamental virological feature creates an exceptional demand for host-derived ribonucleotide triphosphates, which provide the structural units for viral RNA synthesis. Among these, pyrimidines serve as essential nitrogenous bases required for nucleotide biosynthesis.

Pyrimidine metabolism in the central nervous system (CNS) is a complex and highly regulated biological process. It not only supplies essential precursors for nucleic acid synthesis but also plays a profound role in regulating neural development, functional maintenance and responses to injury and disease.^6^, ^7^, ^8^ The CNS maintains pyrimidine nucleotide pools through the coordinated actions of the *de novo* and salvage synthesis pathways.^6^^,^^7^ The *de novo* synthesis pathway relies on a series of enzymes, including dihydroorotate dehydrogenase (DHODH), and involves sequential reactions in the cytoplasm and mitochondria to ultimately produce uridine monophosphate (UMP). It is coupled to the mitochondrial respiratory chain, and its activity is influenced by cellular energy status.^9^ Given that this pathway exhibits elevated activity in the developing brain and under pathological conditions like glioma, it follows that *de novo* synthesis is indispensable for sustaining the cell proliferation.^10^^,^^11^ Accordingly, targeting this metabolic dependency presents a viable therapeutic strategy. DHODH inhibition can selectively deplete the pyrimidine pool in tumor cells, impair ribosomal RNA transcription and induce cell death, while having a lesser impact on normal neural cells that can compensate via the salvage pathway.^11^

In contrast, the adult nervous system depends more heavily on the efficient and energy-saving salvage pathway to maintain pyrimidine pool homeostasis.^8^ This involves the uptake of circulating pyrimidine nucleosides from the blood, followed by their phosphorylation to regenerate nucleotides like uridine triphosphate and cytidine triphosphate (CTP), which support RNA/DNA synthesis and other critical functions.^6^^,^^7^

Pyrimidine metabolism also demonstrates notable cell-type specificity within the CNS. Different neural cells, such as neurons and glia, may exhibit distinct metabolic preference. Supported by the predominant localization of *de novo* pyrimidine biosynthesis enzymes and their mRNA in astrocytes, these cells may constitute the primary site for *de novo* pyrimidine biosynthesis in the brain, whereas mature neurons predominantly rely on the salvage pathway for nucleotides.^7^ However, how JEV infection remodels pyrimidine metabolic programs in different CNS cells remains unclear.

In this study, we analyzed single-cell RNA sequencing (scRNA-seq) data to dissect the pyrimidine metabolic transcriptomic reprogramming induced by JEV infection at single-cell resolution. We systematically mapped the expression of genes involved in pyrimidine biosynthesis, glutamine utilization, and aspartate synthesis across diverse neural and immune cell types. By integrating these transcriptional profiles with functional virological assays, we demonstrate that JEV replication is critically dependent on the *de novo* pyrimidine biosynthesis pathway in neurons. Furthermore, we elucidate that JEV upregulated the glutamine uptake to enhance nitrogen acquisition and upregulates glutamate oxaloacetate transaminase (GOT), the key enzymes of the malate-aspartate shuttle, to secure aspartate production, thereby ensuring a robust supply of precursors for viral RNA genome replication. Our findings provide novel insights into neurotropic virus-host interactions and highlighting potential metabolic vulnerabilities for targeted antiviral intervention.

## 2 Materials and methods

### 2.1 Cells and viruses

Neuro-2a (N2a) (mouse neuroblastoma) and BHK-21 (baby hamster kidney-21) cell lines were cultured in Dulbecco’s modified Eagle medium (DMEM) supplemented with 10 % fetal bovine serum (FBS), 100 U/ml penicillin, and 100 μg/ml streptomycin. The JEV P3 strain was propagated in BHK-21 cells for *in vitro* experiments.

Primary mixed neuron/glia cultures were prepared from the cerebral cortices of neonatal C57BL/6 mice as previously described.^12^ Briefly, dissected cortices were dissociated by trypsinization and mechanical trituration, and cells were plated onto poly-L-lysine-coated plates in Neurobasal medium (Thermo Fisher Scientific, Waltham, USA) supplemented with 2 % FBS, 2 % B-27, 0.5 mM GlutaMAX, 100 U/mL penicillin, and 100 μg/mL streptomycin. Half of the medium was replaced every 3.5 days. Cultures were maintained for 7 days before use and consisted of a mixed population of neurons and glial cells.

### 2.2 Viral titration

Viral titers were determined by plaque assay on BHK-21 cells. Briefly, supernatants from infected cells were collected, serially diluted 10-fold in DMEM, and used to inoculate BHK-21 monolayers. After adsorption at 37°C for 1.5 h, the inoculum was removed, and cells were overlaid with DMEM containing 2 % FBS and 1.5 % carboxymethyl cellulose, followed by incubation at 37°C for 4 to 5 days. Cells were then fixed with 10 % formaldehyde overnight, stained with 0.1 % crystal violet for 2 h, and plaques were counted to calculate viral titer. Viral titers were expressed as PFU/mL.

### 2.3 Reagents and antibodies

Brequinar (BQR, HY-108325), GOT1 inhibitor-1 (GOT1i, HY-122723), L-aspartic acid sodium (HY-N0666C) and V9302 (HY-112683) were purchased from MedChem Express (Monmouth Junction, USA). Uridine (U3003) were purchased from SigmaAldrich (St. Louis, USA) as exogenous nucleotides. L-Alanyl-L-glutamine supplement (200 mM, 100 ×, G4565) for Gln replenishment was purchased from Servicebio (Wuhan, China). Nucleosides (100 ×, PWL089) was purchased from MeilunBio (Dalian, China).

Mouse anti-JEV-E monoclonal antibody (mAb) were prepared in our laboratory. Rabbit anti-DHODH polyclonal antibody (pAb, 14877-1-AP) and anti-solute carrier (SLC) 1A5 pAb (20350-1-AP) were purchased from Proteintech (Wuhan, China). Rabbit anti-GOT1 pAb (A11363), anti-GOT2 mAb (A19245), and anti-β-actin mAb (AC026) were purchased from ABclonal Technology (Wuhan, China). Horseradish peroxidase - conjugated goat anti-mouse IgG (BA1051) and anti-rabbit IgG (BA1055) were purchased from Boster (Wuhan, China). All commercial antibodies were used at concentrations recommended by the manufacturers.

### 2.4 RNA extraction and reverse transcription quantitative polymerase chain reaction (RT-qPCR)

In accordance with the manufacturer’s instructions, the total RNA was extracted from treated samples using TRIzol reagent (Magen, Guangzhou, China). The cDNA was then synthesized from 1 µg of RNA using the ABScript II cDNA First-Strand Synthesis Kit (ABclonal Technology). Quantitative real-time PCR was conducted with the 2 × University SYBR Green Fast qPCR Mix (ABclonal Technology) on a ViiA™ 7 Real-time PCR System (Applied Biosystems, Foster City, USA). The relative expression levels of the targeted mRNA were normalized to that of endogenous gene β-actin within each sample and analyzed using the 2^−ΔΔ^*^ct^* method. The primer sequences used were as follows: JEV-E forward, 5′- TGGTTTCATGACCTCGCTCTC −3′; JEV-E reverse, 5′- CCATGAGGAGTTCTCTGTTTCT −3′; β-actin forward, 5′- CACTGCCGCATCCTCTTCCTCCC −3′; β-actin reverse, 5′- CAATAGTGATGACCTGGCCGT-3′.

### 2.5 Western blotting

Cell lysis was performed using RIPA buffer (Beyotime, Shanghai, China, P0013) containing a protease inhibitor cocktail (TargetMol, Boston, USA, C0001). Protein concentration was determined with the Enhanced BCA Protein Assay Kit (Beyotime, P0010S). Proteins were resolved by sodium dodecyl sulfate–polyacrylamide gel electrophoresis and subsequently transferred to a polyvinylidene fluoride membrane (Millipore) employing a semi-dry transfer system (Mini Trans-Blot Cell, Bio-Rad, Hercules, USA). After blocking with 2 % bovine serum albumin in Tris-buffered saline containing 0.1 % Tween-20 for 2 h, the membranes were incubated overnight at 4°C with specific primary antibodies. Following three washes with Tris-buffered saline containing 0.1 % Tween-20, the membranes were incubated with species-matched horseradish peroxidase-conjugated secondary antibodies for 45 min at room temperature. Signal detection was carried out using an enhanced chemiluminescence substrate (Tanon, Shanghai, China).

### 2.6 Analysis of scRNA-seq data

The scRNA-seq data employed here originated from our earlier research,^13^ which has been deposited in the National Center for Biotechnology Information Gene Expression Omnibus under the accession number GSE237915. We directly utilized the expression data as provided in the original study and the Gene Expression Omnibus entry. The downstream analyses were performed in R (v4.3.0; R Foundation for Statistical Computing, Vienna, Austria), and the expression matrix of metabolic enzymes was exported using the Seurat R package (v 5.3.1). Batch effects were corrected using the Mutual Nearest Neighbors algorithm. Gene expression data were normalized using the LogNormalize method in Seurat. For comparison of pyrimidine metabolism gene expression across groups, differential expression analysis was performed using Seurat’s FindMarkers function with the Wilcoxon rank‑sum test. Adjusted *p* were calculated using Bonferroni correction to control for multiple testing. Cell-type-specific signature genes for pyrimidine metabolism were identified based on fold change (FC) and *z*-score, together with statistical significance. Genes with FC > 1.5, *z*-score > 1.5, and adjusted *p* < 0.05 were selected as signature genes.

For infiltrating immune cells, including T cells, monocytes/macrophages (Monocytes/MΦ), and NK cells, analyses were focused on JEV-N versus JEV-P comparisons because mock controls were unavailable due to insufficient cell numbers. To characterize neuronal responses across different infection intensities, JEV-P neurons were further stratified into three groups based on viral genomic expression levels: viral genome-low (JEV-L; log₂[JEV expression] < 3), viral genome-medium (JEV-M; 3 ≤ log₂[JEV expression] ≤ 8), and viral genome-high (JEV-H; log₂[JEV expression] > 8).

For analytical visualization, the expression profiles of metabolic enzyme genes were presented in two forms: a heatmap, generated using the ComplexHeatmap R package (v2.24.1), to compare expression levels across cell clusters; and violin plots, created with the ggplot2 R package (v 4.0.0), to illustrate the probability density and distribution of expression for specific genes of interest. All visualizations were generated in R (v4.3.0; R Foundation for Statistical Computing, Vienna, Austria).

### 2.7 Inhibitor treatment *in vitro*

N2a cells were infected with JEV at a multiplicity of infection (MOI) of 1 or 5 in serum-free medium. After a 1.5-h adsorption period, the inoculum was replaced with fresh DMEM containing 2 % FBS. Inhibitors, at doses predetermined by a Cell Counting Kit-8 cytotoxicity assay (SuperKine™ Maximum Sensitivity Cell Counting Kit-8; Abkkine, Wuhan, China, BMU106), were then added to the culture medium. Supernatants and cells were harvested at designated time points for assessing viral titer by plaque assay and viral RNA abundance by RT-qPCR.

### 2.8 Plasmid construction and transfection

The full-length mouse cytidine/uridine monophosphate kinase 2 (CMPK2) coding sequence was amplified by PCR and cloned into the p3 × Flag-CMV-10 vector to generate Flag-CMPK2. The construct was verified by Sanger sequencing. N2a cells were transfected with Flag-CMPK2 or empty vector control using Lipofectamine 3000 (Invitrogen) according to the manufacturer's instructions. At 24 h post-transfection, cells were infected with JEV (MOI = 5) for functional assays. Overexpression efficiency was confirmed by immunoblotting with anti-Flag antibody (prepared in our lab).

### 2.9 Statistical analysis

All experimental procedures were conducted with at least three independent biological replicates. Statistical analyses were performed with GraphPad Prism (Version 9.0, GraphPad Software, San Diego, USA). Prior to parametric analyses, data distribution was evaluated using the Shapiro-Wilk test. All datasets passed normality testing (*p* > 0.05), and therefore Student’s *t*-test (for two-group comparisons) or analysis of variance (ANOVA; for multiple groups) was applied. If any dataset had violated the normality assumption, the corresponding non-parametric test (Mann-Whitney *U* test or Kruskal-Wallis test) would have been used. The thresholds for statistical significance are defined as: *p < 0.05, **p < 0.01, ***p < 0.001, and ****p < 0.0001; non-significant differences are abbreviated as “ns”.

## 3 Results

### 3.1 Systematic profiling reveals cell-type-specific metabolic programming of pyrimidine metabolism in mouse brain

**Fig. 1:**
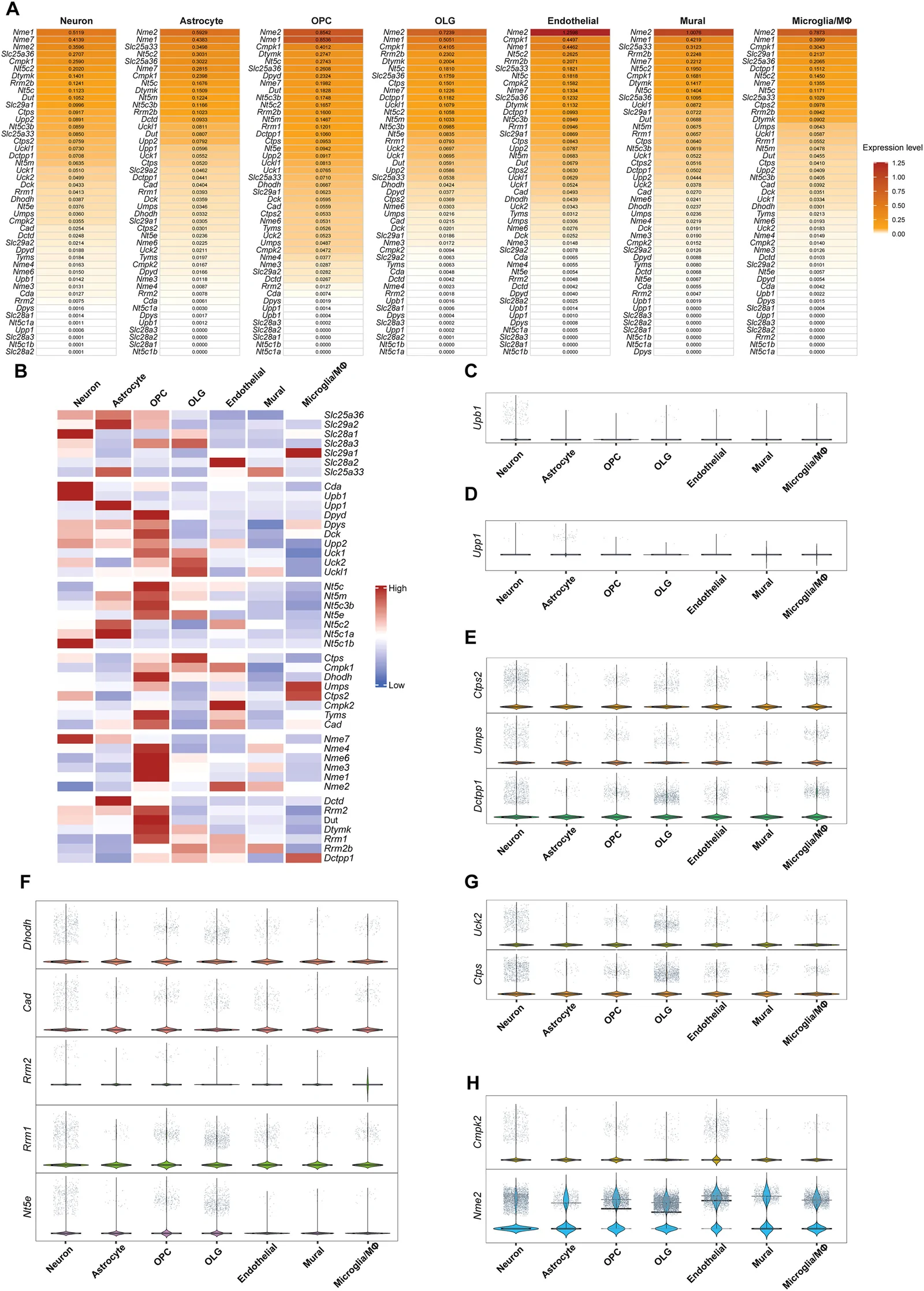
Cell-type-specific metabolic program of pyrimidine metabolism in the normal mouse brain. (A) Heatmap depicting the expression rank of core pyrimidine metabolism genes across seven major cell types isolated from the normal mouse brain. *Nme1* and *Nme2* are universally the most abundant transcripts, establishing a shared baseline for nucleotide triphosphate homeostasis. (B) Comparative heatmap highlighting cell-type-specific deviations from the core metabolic program shown in (A). Each column represents a cell type, with color intensity indicating the average expression across all types. (C–H) Violin plots illustrating the distinct expression signatures of key metabolic enzymes for each major cell type: Neurons (C), astrocytes (D), Microglia/ MΦ (E), oligodendrocyte precursor cells (F), mature oligodendrocytes (G), endothelial cells (H). Genes shown met adjusted *p* < 0.05, fold change > 1.5, and *Z*‑score > 1.5 in the highlighted cell type compared to other cell types. Data are derived from single‑cell RNA sequencing of brain cells from Mock mice (*n* = 3). *Abbreviations*: Microglia/MΦ, microglia/macrophage; OLG, oligodendrocyte; OPC, oligodendrocyte precursor cells.

To elucidate the cell-type-specific metabolic foundations of pyrimidine nucleotide homeostasis in the CNS, we analyzed the expression profiles of key genes involved in pyrimidine metabolism (Supplementary Table S1) across seven major cell types isolated from the normal mouse brain: Neurons, astrocytes, oligodendrocytes, oligodendrocyte precursor cells (OPCs), the microglia/macrophage (Microglia/MΦ) population, and endothelial cells with mural cells, based on our previous single-cell transcriptome data.^13^ Despite the low overall expression of many individual genes, a common core program of nucleotide metabolism was found to be ubiquitously active across all cell types (Fig. 1A). This program is characterized by the predominant expression of nucleoside diphosphate kinases (NME) 1 and NME2, which were the most abundant transcripts overall, highlighting the universal priority of maintaining nucleotide triphosphate pools. The enzymes CMPK1 and deoxythymidylate kinase, constitutively expressed, provide a foundational route for pyrimidine synthesis. Furthermore, the 5′-nucleotidases NT5C and NT5C2, alongside the mitochondrial transporters SLC25A33 and SLC25A36 and the equilibrative transporter SLC29A1, serve as integral nodes within this shared network. This collective profile establishes the essential baseline for pyrimidine nucleotide homeostasis in all brain cells, upon which cell-type-specific specializations are built.

Comparative analysis revealed a pronounced cell-type-specific deviations from the core metabolic program (Fig. 1B), with each major brain cell type expressing a distinct enzymatic repertoire that may align precisely with its core physiological function (Fig. 1C–H). Neurons exhibited a distinct pyrimidine catabolic signature, characterized by markedly high expression of UPB1 (β-ureidopropionase). This upregulation suggests that pyrimidine nucleotide breakdown may serve a dual purpose: Not only for nucleotide pool maintenance but also to channel carbon skeletons into the tricarboxylic acid (TCA) cycle, thereby supporting the high energy demands of post-mitotic neurons (Fig. 1C). In complementary fashion, astrocytes displayed a profile optimized for nucleotide salvage and precursor provision, highlighted by the preferential expression of UPP1 (uridine phosphorylase 1). This function is integral to their role as key regulators of brain metabolic homeostasis, facilitating the recycling of nutrients and clearance of metabolic waste (Fig. 1D). The Microglia/MΦ population exhibited a dual signature tailored for immune surveillance. It showed the highest enrichment for dCTP pyrophosphatase 1 , a key enzyme for deoxyribonucleoside triphosphate pool sanitation and genome stability, alongside elevated expression of *de novo* synthesis enzymes CTP synthase (CTPS) 2 and uridine monophosphate synthase (UMPS), indicating a metabolism prepared for both nucleotide pool and genomic integrity, alongside a rapid anabolic response upon activation (Fig. 1E). Within the oligodendrocyte lineage, a clear developmental transition was evident. OPCs exhibited a robust anabolic program characterized by elevated expression of key enzymes involved in *de novo* pyrimidine biosynthesis, including carbamoyl-phosphate synthetase 2, aspartate transcarbamylase, and dihydroorotase (CAD) and DHODH, together with ribonucleotide reductase regulatory subunit M1 and M2, which support deoxyribonucleotide production for DNA replication and proliferation. OPCs also showed high expression of 5′-nucleotidase ecto, suggesting enhanced purinergic signaling potential (Fig. 1F). In contrast, mature oligodendrocytes (OLG) shifted towards a maintenance-oriented profile, retaining high expression of (CTPS and uridine-cytidine kinase (UCK) 2. This dual capacity for *de novo* CTP synthesis (via CTPS) and pyrimidine salvage (via UCK2) ensures a sustained supply of nucleotide precursors for phospholipid biosynthesis, supporting the turnover of myelin membranes (Fig. 1G). Finally, endothelial cells exhibited a specialized profile with unique co-expression of NME2 and CMPK2. This molecular signature suggests enhanced regulation of nucleotide phosphorylation and homeostasis, potentially supporting the metabolic requirements associated with endothelial cellular functions and vascular remodeling (Fig. 1H). Collectively, these findings reveal distinct cell type-specific pyrimidine metabolic programs in the normal mouse brain.

### 3.2 JEV infection reprograms pyrimidine biosynthesis in a cell type-specific manner in mouse brain

**Fig. 2:**
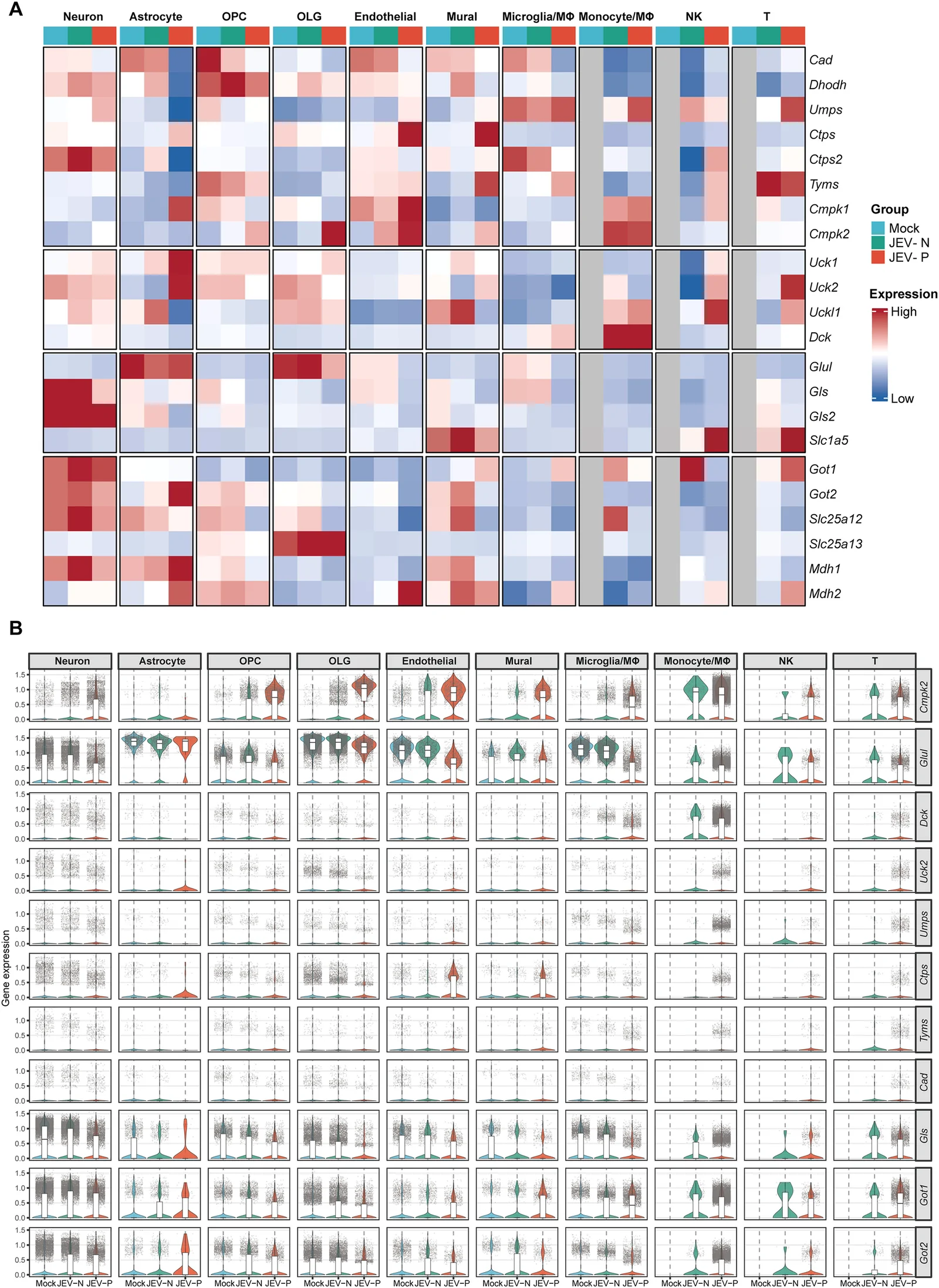
JEV infection reprograms pyrimidine metabolism in a cell-type-specific manner. (A) Heatmap depicting the expression profiles of key enzymes involved in pyrimidine biosynthesis and the associated glutamine and aspartate metabolism across major brain cell populations under three conditions: Mock-infected controls (Mock), JEV-negative (JEV‑N) cells from infected mice, and JEV-positive (JEV‑P) cells. For infiltrating immune cells (T cells, Monocytes/Macrophages, NK cells), only JEV‑P vs. JEV‑N comparisons are shown due to the absence of mock controls. (B) Violin plots illustrating cell‑type‑specific expression changes of selected metabolic genes in response to JEV infection. Data are derived from single‑cell RNA sequencing of brain cells from JEV‑infected (*n* = 5) and mock‑infected (*n* = 3) mice. Differential expression between groups was determined by Wilcoxon rank‑sum test as implemented in Seurat’s FindMarkers function with Bonferroni correction. Genes with adjusted *p* < 0.05 in at least one pairwise comparison were considered statistically significant. *Abbreviations*: JEV, Japanese encephalitis virus; Microglia/MΦ, microglia/macrophage; Mock, mock-infected controls; OLG, oligodendrocyte; OPC, oligodendrocyte precursor cells.

To further investigate the impact of JEV infection on cellular pyrimidine biosynthesis within the CNS, we analyzed the expression profiles of key enzymes involved in pyrimidine biosynthesis across major cell populations. Comparative analysis of JEV genomic RNA-positive (JEV-P) cells, JEV-negative (JEV-N) cells from infected mice, and cells from mock-infected controls (Mock) revealed profound and cell-type-specific alterations in the expression of genes governing both *de novo* and salvage pyrimidine biosynthesis pathways (Fig. 2A). In most examined cell types, JEV-P cells exhibited upregulation of pyrimidine biosynthesis enzymes compared to their JEV-N and Mock counterparts, suggesting an infection-driven increase in nucleotide demand (Fig. 2A, B).

This metabolic reprogramming was particularly striking in vascular and glial compartments. Endothelial cells (EC) demonstrated significant induction of CMPK2 (adjusted *p* < 0.0001, log₂ FC = 3.64) and CTPS (adjusted *p* < 0.0001, log₂ FC = 2.64) in JEV-P cells compared to Mock. Mural cells also exhibited significant upregulation of CMPK2 (adjusted *p* < 0.0001, log₂ FC = 5.96) and CTPS (adjusted *p* < 0.0001, log₂ FC = 2.20). Among glial cells, oligodendrocytes showed the most striking induction of CMPK2 (adjusted *p* < 0.0001, log₂ FC = 8.13). OPCs (adjusted *p* < 0.0001, log₂ FC = 4.67), Neurons (adjusted *p* < 0.0001, log₂ FC = 3.99) and Microglia/MΦ (adjusted *p* < 0.0001, log₂ FC = 5.69) also displayed significant upregulation of CMPK2, suggesting a common metabolic signature across diverse cell populations during JEV infection (Fig. 2A, B).

Given that CMPK2 was one of the most prominently upregulated genes across multiple cell types in our scRNA-seq analysis, we sought to determine its functional impact on JEV replication. While CMPK2 is an interferon-stimulated gene with reported antiviral activity, its role in JEV infection has not been previously characterized. We transfected N2a cells with Flag-tagged CMPK2 or empty vector control and confirmed robust overexpression by immunoblotting (Supplementary Fig. S1). CMPK2 overexpression significantly reduced intracellular viral protein levels at 18–24 hours post-infection (hpi) compared to the vector control (Supplementary Fig. S1), demonstrating an antiviral effect.

In T cells, pyrimidine synthesis enzymes such as UCK2 and UMPS showed a trend toward upregulation in JEV‑P cells (log₂ FC = 1.00 and 0.64, respectively), though these differences did not reach statistical significance after multiple testing correction (adjusted *p* = 1.000). Similarly, in Monocytes/MΦ, CTPS (log₂ FC = 2.00, adjusted *p* = 1.000) and UMPS (log₂ FC = 0.54, adjusted *p* = 1.000) exhibited numerical increases in JEV‑P relative to JEV‑N. In NK cells, several *de novo* synthesis enzymes such as CTPS (log₂ FC = 0.68), UCK1 (log₂ FC = 1.83), UCK2 (log₂ FC = 1.38), and TYMS (log₂ FC = 0.88) showed numerical upregulation in JEV‑P; however, none of these differences reached statistical significance, likely due to the low number of NK cells captured. CAD and GOT1 in NK cells also showed numerical decreases in JEV‑P (log₂ FC = −0.32 and −1.49, respectively). (Fig. 2A, B).

Glutamine serves as a crucial nitrogen donor for *de novo* nucleotide synthesis. Upon JEV infection, multiple cell types exhibited significant downregulation of key genes in the glutamine-glutamate cycle. The glutaminase (GLS) was significantly reduced in JEV-P neurons compared to Mock (adjusted *p* < 0.0001, log₂ FC = −1.25), and glutamine synthetase (GLUL) was also significantly downregulated (adjusted *p* = < 0.0001, log₂ FC = −1.10). Similar significant reductions in GLS and GLUL were observed in EC, Microglia/MΦ, and oligodendrocytes in their JEV-P states (Fig. 2A, B, Supplementary Table S2). Mural cells exhibited a trend toward GLS downregulation (unadjusted *p* = < 0.0001, adjusted *p* = 0.41), while GLUL expression remained unchanged. Among infiltrating immune cells, T cells displayed significant upregulation of the glutamine transporter SLC1A5 in JEV-P (log₂ FC = 1.67, adjusted *p* < 0.001). In NK cells, SLC1A5 also showed a numerical increase in JEV-P (log₂ FC = 1.25, adjusted *p* = 1.000). These findings reveal a broad reprogramming of glutamine metabolism across CNS and immune cells during JEV infection.

Enzymes linking aspartate to nucleotide metabolism also showed cell-type-specific alterations upon JEV infection. GOT1 was significantly upregulated in JEV‑P compared to Mock in Microglia/MΦ (adjusted *p* < 0.0001, log₂ FC = 0.83), mural cells (adjusted *p* < 0.0001, log₂ FC = 0.81), and EC (adjusted *p* < 0.0001, log₂ FC = 0.07). In neurons, GOT1 was significantly upregulated in JEV‑P compared to Mock (adjusted *p* < 0.0001, log₂ FC = 0.13), while GOT2 was significantly downregulated (adjusted *p* < 0.0001, log₂ FC = –0.20). GOT2 was also significantly downregulated in Microglia/MΦ (adjusted *p* < 0.0001, log₂ FC = −0.24) (Fig. 2A, B). These transcriptional changes suggest that JEV infection broadly upregulates GOT1 expression in CNS cells, which could enhance aspartate availability for pyrimidine biosynthesis. Collectively, these data reveal JEV-induced pyrimidine metabolic reprogramming, with the strongest activation in JEV-P cells.

### 3.3 JEV replication depends on *de novo* pyrimidine biosynthesis

**Fig. 3:**
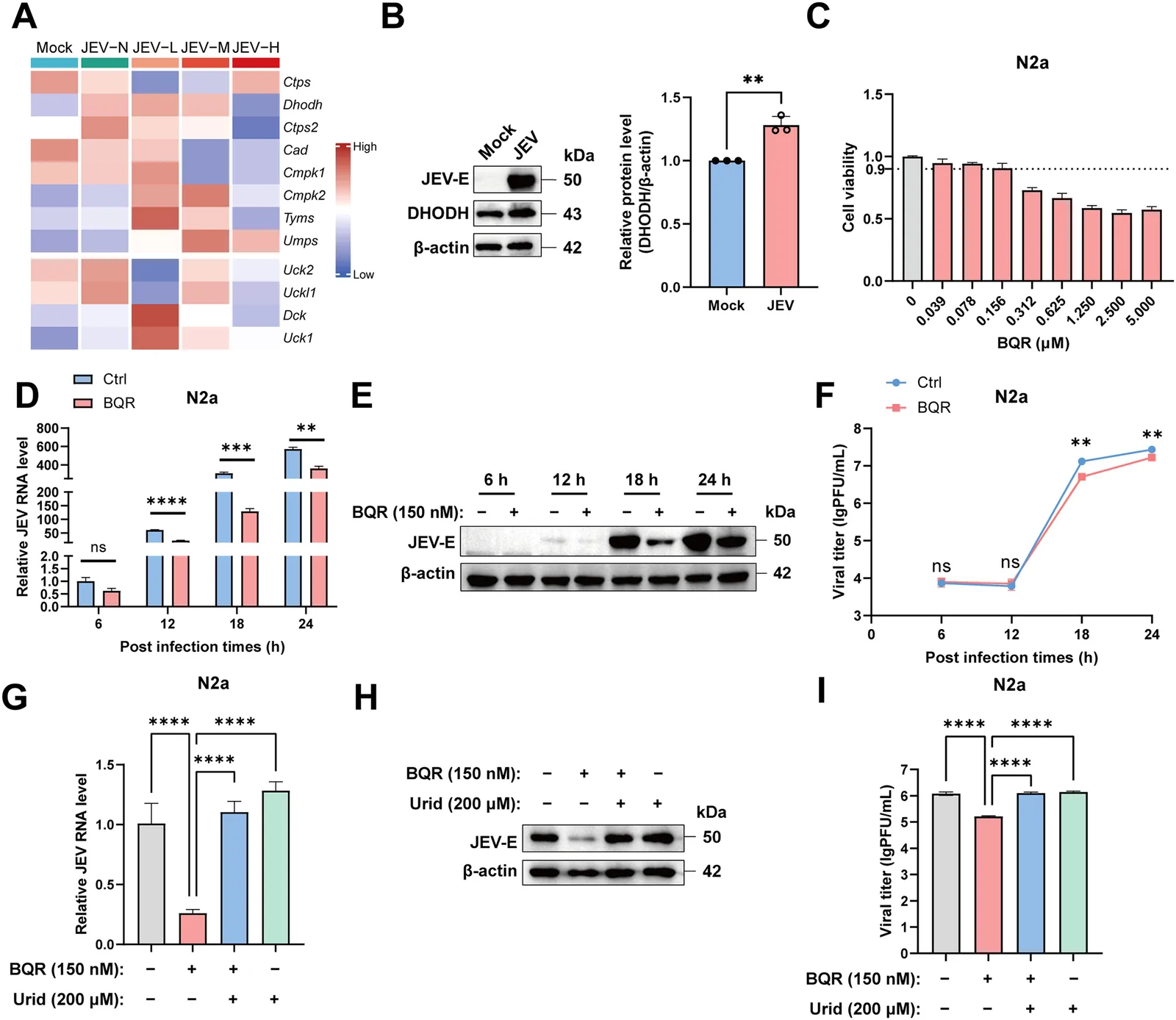
JEV replication depends on *de novo* pyrimidine biosynthesis. (A) Heatmap of key pyrimidine synthesis enzyme genes in neurons, stratified by JEV infection intensity: Mock, JEV-Negative (JEV-N), JEV-Low (JEV-L), JEV-Medium (JEV-M), and JEV-High (JEV-H). Data are derived from single‑cell RNA sequencing of neurons from JEV‑infected (*n* = 5) and mock‑infected (*n* = 3) mice. (B) JEV infection upregulates the protein expression of key *de novo* pyrimidine biosynthesis (DNPB) enzymes DHODH. Western blot analysis of DHODH protein expression in N2a cells infected with JEV (MOI = 1) for 48 h. β-actin serves as a loading control. Blots are representative of three independent experiments. Bar graph shows densitometric quantification of DHODH levels normalized to β-actin (*n* = 3). (C) Cell viability assay (Cell Counting Kit-8) of N2a cells treated with indicated amount DHODH inhibitor brequinar (BQR) for 24 h. (D–F) BQR treatment significantly reduces JEV replication in N2a cells. N2a cells were infected with JEV at an MOI of 5, followed by treatment with either 150 nM BQR at 1.5 hpi. The viral RNA level (D) and E protein (E), as well as the viral titer (F) at 6, 12, 18, and 24 hpi, were determined using RT-qPCR, immunoblotting, and plaque assay, respectively. (G–I) Supplementation with 200 μM uridine restores JEV replication suppressed by BQR. N2a cells infected with 5 MOI JEV were treated with either BQR or dimethyl sulfoxide, with or without supplementation of uridine at 1.5 hpi. The viral RNA level (G), Viral E protein (H) and viral titers (I) were measured at 18 hpi. Data are presented as mean ± standard error of the mean from 3 independent biological replicates (*n* = 3). Significance was determined using two-tailed unpaired Student’s t-test (B), one-way (G and I) or two-way ANOVA (D and F) (**p < 0.01, ***p < 0.001; ****p < 0.0001; non-significant differences are abbreviated as “ns”). *Abbreviations*: ANOVA, analysis of variance; BQR, brequinar; DHODH, dihydroorotate dehydrogenase; DNPB, *de novo* pyrimidine biosynthesis; JEV, Japanese encephalitis virus; Mock, mock-infected controls; MOI, multiplicity of infection; N2a, Neuro-2a; RT-qPCR, reverse transcription quantitative polymerase chain reaction.

Given that JEV is a neurotropic virus with neurons as its primary cellular targets, we focused our analysis on the impact of JEV infection on pyrimidine synthesis in neurons with scRNA-seq data. Heatmap analysis revealed significantly elevated expression of genes encoding key enzymes UMPS (adjusted *p* < 0.0001, log₂ FC = 0.50) and DHODH (adjusted *p* < 0.0001, log₂ FC = 0.30) in the *de novo* pyrimidine biosynthesis pathway in JEV-M neurons compared to Mock (Fig. 3A).

Then, we employed the N2a cell line as an *in vitro* model to mechanistically dissect the functional importance of *de novo* pyrimidine biosynthesis in neurons. Consistent with this transcriptional profile, the protein expression of DHODH, a pivotal enzyme in this pathway, was also markedly upregulated at 48 hpi in N2a model (Fig. 3B). To assess whether *de novo* pyrimidine biosynthesis pathway contributes to JEV replication, we pharmacologically inhibited this pathway using 150 nM brequinar (BQR) (Fig. 3C), an inhibitor of DHODH. BQR treatment significantly reduced JEV replication in N2a cells, as indicated by reduced levels of viral RNA, protein expression, and infectious virion production (Fig. 3D−F). Moreover, the supplementation with 200 μM uridine, a downstream metabolite of DHODH, in the culture medium restored JEV replication under BQR-mediated inhibition (Fig. 3G−I). These coordinated suppressive effects underscore the essential role of *de novo* pyrimidine biosynthesis in sustaining JEV replication cycles, consistent with the upregulation of the key enzyme genes in pyrimidine metabolism pathway observed in JEV-highly infected neurons (Fig. 3A).

To confirm these findings in a more physiologically relevant system, we validated the key observations in primary mouse brain-derived mixed neuron/glia cultures (Supplementary Fig. S2A). JEV infection (MOI = 1) upregulated DHODH protein at 24–36 hpi in these primary cultures (Supplementary Fig. S2B), mirroring the N2a results. Importantly, BQR treatment significantly suppressed JEV replication at 18 hpi, and this inhibition was fully rescued by exogenous uridine supplementation (Supplementary Fig. S2C), demonstrating that the dependence on *de novo* pyrimidine synthesis is not restricted to the N2a cell line but is also operative in primary CNS cells.

### 3.4 JEV infection depends on exogenous glutamine uptake

**Fig. 4:**
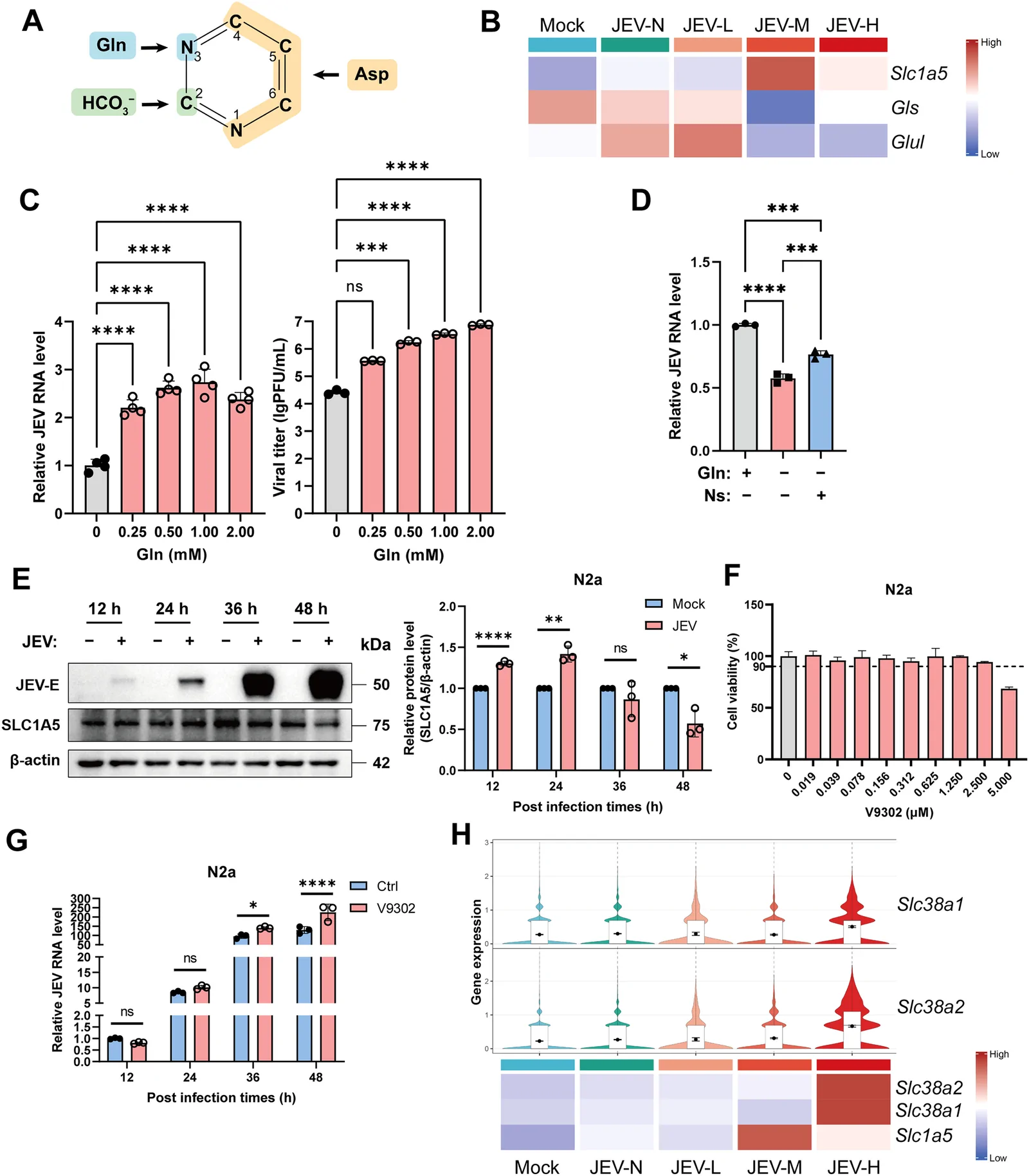
JEV infection depends on exogenous glutamine uptake in neurons. (A) Schematic of the pyrimidine ring, highlighting the origins of nitrogen and carbon atoms from glutamine and aspartate. (B) Single-cell heatmap showing expression dynamics of glutamine metabolism genes (*Gls, Glul* and *Slc1a5*) in neurons across Mock, JEV-N, JEV-L, JEV-M, and JEV-H groups. Data are derived from single‑cell RNA sequencing of neurons from JEV‑infected (*n* = 5) and mock‑infected (*n* = 3) mice. (C) JEV replication in N2a cells is impaired in glutamine-depleted medium and restored in a concentration-dependent manner with glutamine supplementation. Following infection with JEV (MOI = 1), N2a cells were cultured for 36 h in glutamine-depleted medium containing various concentrations of glutamine. (D) Partial rescue of JEV replication upon supplementation with exogenous nucleosides under conditions of glutamine deprivation. (E) Time-course analysis of SLC1A5 protein expression upon JEV infection. N2a cells were infected with JEV (MOI = 1) or mock-infected. Cells were harvested at the indicated time points and subjected to immunoblotting for SLC1A5 and β-actin. Relative protein expression levels were quantified and normalized to β-actin. (F) Cell viability of N2a cells treated with the SLC1A5 inhibitor V9302. N2a cells treated with indicated amount V9302 for 48 h. (G) Time-course analysis of JEV RNA levels in N2a cells treated with the SLC1A5 inhibitor V-9302 (2.5 μM) or vehicle control. N2a cells were infected with JEV at an MOI of 1, followed by treatment with V-9302 at 1.5 hpi. Viral RNA was quantified by RT-qPCR at indicated time points post-infection. (H) Heatmap and violin plots depicting the expression of alternative glutamine transporter genes (*Slc38a1, Slc38a2*) in neurons from scRNA-seq data. Data are derived from single‑cell RNA sequencing of neurons from JEV‑infected (*n* = 5) and mock‑infected (*n* = 3) mice. For experiments except the scRNA-seq analysis, data are presented as mean ± standard error of the mean from 3 independent biological replicates (*n* = 3). Significance was determined using two-tailed unpaired Student’s *t*-test (E), one-way (C, D) or two-way ANOVA (G) (*p < 0.05, **p < 0.01, ***p < 0.001; ****p < 0.0001; ns, not significant). *Abbreviations*: JEV, Japanese encephalitis virus; Mock, mock-infected controls; MOI, multiplicity of infection; N2a, Neuro-2a; RT-qPCR, reverse transcription quantitative polymerase chain reaction; scRNA-seq, single-cell RNA sequencing.

The reliance of viral replication on the host *de novo* pyrimidine biosynthesis pathway creates a heightened demand for its metabolic precursors. As a key amino acid, glutamine provides essential carbon and nitrogen, and its γ-nitrogen can be directly utilized for nucleotide biosynthesis (Fig. 4A). Our scRNA-seq data revealed that the gene expression of GLS and GLUL was downregulated with increasing infection intensity (Fig. 2A and 4B). In contrast, mRNA levels of the key glutamine transporter SLC1A5 were numerically upregulated (log₂ FC = 1.31, unadjusted *p* < 0.0001, adjusted *p* = 0.059) in JEV-M cells compare to Mock (Fig. 4B), suggesting that JEV replication in neurons likely depends on the uptake of exogenous glutamine rather than its intracellular synthesis. This glutamine dependence was further confirmed by functional assays, as evidenced by the severe impairment of JEV replication in N2a cells cultured in glutamine-depleted medium and its concentration-dependent restoration upon glutamine supplementation (Fig. 4C). Notably, supplementing exogenous nucleosides partially rescued the replication defects caused by glutamine deprivation (Fig. 4D).

Given that SLC1A5 is widely recognized as the primary transporter mediating cellular glutamine uptake,^14^ we next examined SLC1A5 protein expression in infected N2a cells and observed a time-dependent dynamic characterized by a significant increase at 12–24 hpi followed by a gradual decline by 36–48 hpi (Fig. 4E). This expression pattern paralleled the transcriptional changes seen in infected mouse brains (Fig. 4B), where SLC1A5 expression upregulation peaked in moderately infected neurons and was attenuated in highly infected neurons. However, functional inhibition of SLC1A5 via the specific inhibitor V9302 (Fig. 4F) only induced a numerical decrease in JEV RNA replication level in early infection (12 hpi), and this change did not reach statistical significance. Unexpectedly, at later time points (36–48 hpi), V‑9302 treatment significantly enhanced viral replication (Fig. 4G). These results suggest that while JEV replication depends on exogenous glutamine, SLC1A5 is not the sole mediator of glutamine uptake in infected cells. Rather, prolonged blockade of SLC1A5 may trigger compensatory upregulation of alternative glutamine transporters, ultimately sustaining or even enhancing glutamine supply for viral replication. Re‑examination of our scRNA‑seq data revealed that, in the JEV‑high neuronal group where SLC1A5 expression decreased, the expression of alternative glutamine transporters SLC38A1 and SLC38A2 was elevated (Fig. 4H). This expression pattern suggests a potential compensatory response that may help maintain glutamine uptake under conditions of SLC1A5 attenuation.

### 3.5 JEV upregulates GOT-mediated aspartate synthesis to support viral replication

**Fig. 5:**
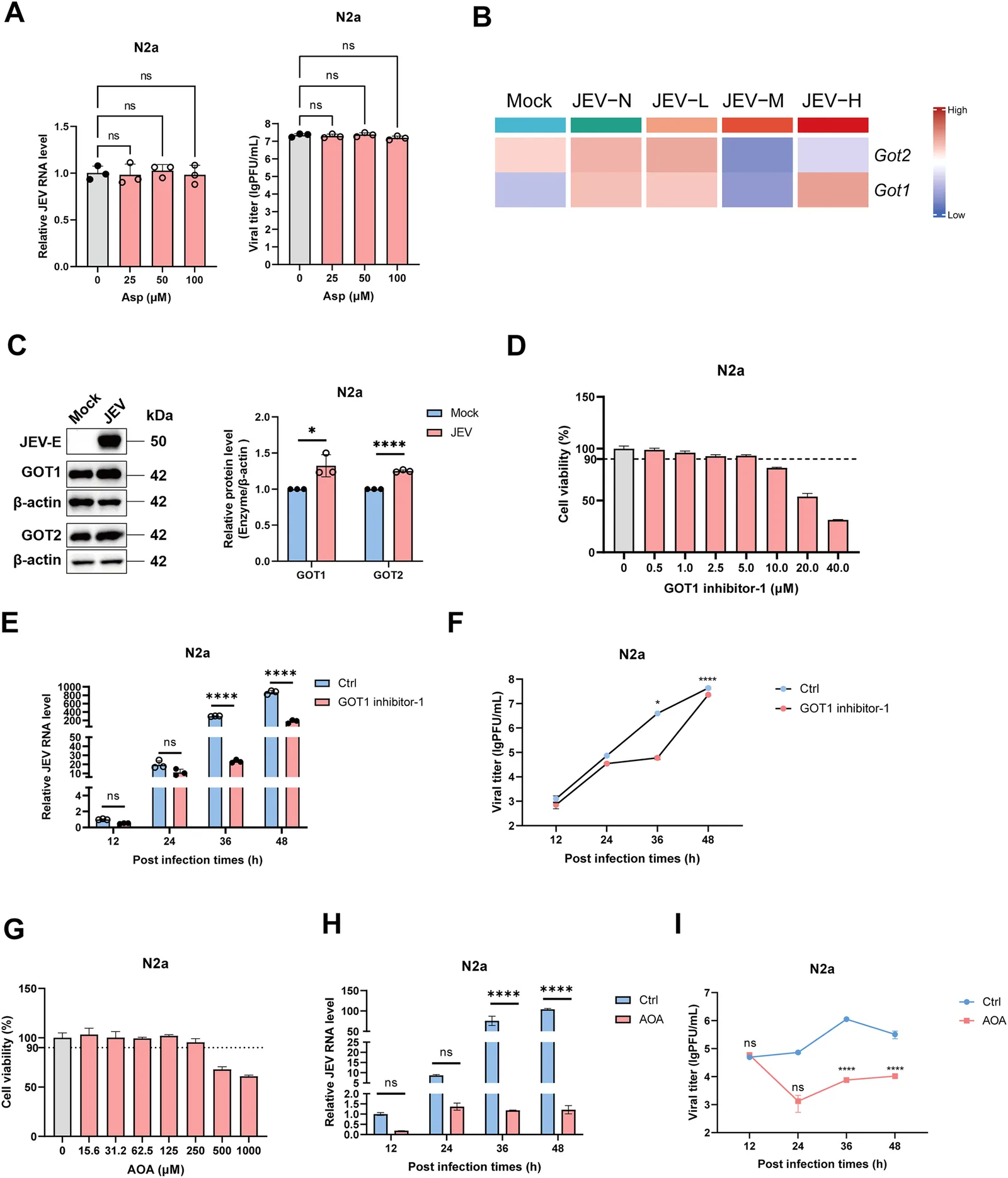
JEV activates the malate-aspartate shuttle for *de novo* aspartate synthesis. (A) Exogenous aspartate supplementation fails to enhance JEV replication in N2a cells. N2a cells were infected with JEV (MOI = 1) and cultured in standard DMEM supplemented with graded concentrations of aspartate for 36 h. Viral replication was assessed by RT-qPCR and plaque assay (*n* = 3). (B) Single-cell RNA sequencing analysis shows *Got1* and *Got2* expression in neurons across infection groups. Heatmap depicts the mean expression levels. Significant upregulation of *Got1* is evident in JEV-H cells compared to Mock group. Data are derived from single‑cell RNA sequencing of neurons from JEV‑infected (*n* = 5) and mock‑infected (*n* = 3) mice. (C) JEV infection upregulates GOT1 and GOT2 protein expression. N2a cells were infected with JEV (MOI = 1) or mock-treated. Cells were harvested at indicated time points and analyzed by immunoblotting for GOT1, GOT2, and β-actin. Representative blots from three independent experiments are shown. Protein levels were quantified and normalized to β-actin (*n* = 3). (D) Cell viability of N2a cells treated with the GOT1i. N2a cells treated with indicated amount GOT1i for 48 h. (E-F) Pharmacological inhibition of GOT1 activity significantly suppresses JEV replication. N2a cells were infected with JEV (MOI = 1) and treated with 5 μM GOT1i or vehicle at 1.5 hpi. Viral replication was significantly reduced at 36–48 hpi, as measured by viral RNA levels (E) and infectious titer (F). (G) Cell viability of N2a cells treated with AOA. N2a cells treated with indicated amount for 48 h. (H-I) AOA treatment suppresses JEV replication in N2a cells. N2a cells were infected with JEV (MOI = 1) and treated with AOA (250 μM) at 1.5 hpi. Viral RNA levels (H) were quantified by RT-qPCR, and supernatant viral titers (I) were determined by plaque assay at 12–48 hpi (*n* = 3). For experiments except the scRNA-seq analysis, data are presented as mean ± standard error of the mean from 3 independent biological replicates (*n* = 3). Significance was determined using two-tailed unpaired Student’s *t*-test (C), one-way (A) or two-way ANOVA (E-F and H-I) (*p < 0.05, ****p < 0.0001; ns, not significant). *Abbreviations*: AOA, aminooxyacetic acid; DMEM, Dulbecco’s modified Eagle medium; JEV, Japanese encephalitis virus; Mock, mock-infected controls; MOI, multiplicity of infection; N2a, Neuro-2a; RT-qPCR, reverse transcription quantitative polymerase chain reaction.

Given that aspartate provides the carbon backbone for pyrimidine ring formation (Fig. 4A), we next examined whether JEV modulates aspartate metabolism to meet the increased precursor demands. To test this, JEV-infected N2a cells were cultured with increasing concentrations of exogenous aspartate. Supplementation did not significantly enhance viral RNA levels or infectious virion production (Fig. 5A), suggesting that intracellular aspartate synthesis, rather than exogenous uptake, is the primary route for satisfying the virus-induced demand.

We next investigated the malate–aspartate shuttle, a source of aspartate production mediated by GOT1/GOT2. Analysis of scRNA-seq data revealed that, in JEV-high neurons, GOT1 transcripts were significantly upregulated (adjusted *p* < 0.0001, log₂ FC = 0.39) compared to Mock controls, whereas GOT2 exhibited a modest but statistically significant downregulation (adjusted *p* < 0.0001, log₂ FC = −0.09) (Fig. 5B). However, JEV infection markedly increased both GOT1 and GOT2 protein expression in N2a cells, with a pronounced upregulation observed at 48 hpi (Fig. 5C).

To test the functional contribution of this pathway, we pharmacologically inhibited GOT1 enzymatic activity using GOT1 inhibitor-1 (GOT1i). A Cell Counting Kit-8 cytotoxicity assay confirmed that GOT1i at concentrations up to 5 μM did not significantly affect N2a cell viability over 48 h (Fig. 5D). Treatment with 5 μM GOT1i at 1.5 hpi significantly reduced JEV RNA levels and infectious virion production at 36–48 hpi (Fig. 5E, F), indicating that GOT1-mediated aspartate synthesis contributes to efficient JEV replication. We next sought to corroborate these findings using aminooxyacetic acid (AOA) (Fig. 5G), a broad-spectrum inhibitor of GOT1/2 transaminase activity. AOA treatment significantly suppressed both intracellular JEV RNA levels and supernatant viral titers at 36–48 hpi (Fig. 5H, I). The consistent inhibition observed with both the GOT1i and AOA reinforces the conclusion that transaminase activity, and specifically GOT1-mediated aspartate biosynthesis, is critical for sustaining JEV replication in neuronal cells.

We further validated the role of the GOT in primary mouse brain-derived mixed neuron/glia cultures. Consistent with the N2a data, JEV infection led to a clear upregulation of GOT1 protein at 36 hpi, whereas GOT2 expression remained unchanged in these primary cultures (Supplementary Fig. S2D). This pattern is also similar to the neuronal scRNA‑seq data, in which GOT1 was selectively upregulated in JEV‑high neurons while GOT2 was modestly downregulated. This suggests that GOT1 may be the primary transaminase responsive to JEV-induced metabolic demand in CNS cells. Functionally, treatment with AOA significantly reduced JEV replication at 24 hpi (Supplementary Fig. S2E), confirming that GOT-mediated aspartate synthesis is critical for JEV propagation in primary CNS cells.

## 4 Discussion

Pyrimidine metabolism plays a fundamental role in maintaining host cellular homeostasis and supporting RNA virus replication. Beyond JEV, several viruses significantly enhance the host *de novo* pyrimidine biosynthesis. Human cytomegalovirus infection channels newly synthesized pyrimidines toward viral glycoprotein glycosylation,^15^ while enterovirus 71 directly activates the CAD enzyme via its 2C protein.^16^ Notably, inhibiting key enzymes in this pathway, such as DHODH, effectively suppresses the replication of a broad spectrum of viruses, including Ebola virus,^17^ dengue virus,^18^ influenza viruses^19^ and African swine fever virus.^20^ While the dependence of RNA viruses on *de novo* pyrimidine biosynthesis has been recognized for multiple viruses, the fundamental novelty of the current study resides in the single-cell resolution mapping of pyrimidine metabolic rewiring across diverse CNS cell types. Here, we provide this cell-type-resolved map of pyrimidine metabolism in the brain and reveals how JEV dynamically rewires this network, offering a resource for understanding metabolic vulnerabilities during neurotropic viral infection.

The pronounced upregulation of CMPK2 in multiple cell types was one of the most striking transcriptional changes in JEV-infected brains. CMPK2 is a mitochondrial UMP‑CMP kinase that contributes to pyrimidine nucleotide supply. However, it is also a well‑characterized interferon‑stimulated gene induced by RIG‑I/MDA5‑dependent signaling and has been reported to exert antiviral activity against Zika virus and Newcastle disease virus.^21^, ^22^, ^23^ In N2a cells, overexpression of Flag-CMPK2 significantly suppressed JEV replication, indicating that CMPK2 upregulation during JEV infection likely represents an antiviral host response rather than a pro-viral metabolic adaptation. Although its kinase activity could theoretically aid pyrimidine provision, the net antiviral effect observed under overexpression conditions suggests that its immune-related functions dominate in this context. Definitive dissection of these dual roles will require future experiments with kinase dead mutants or cell type specific knockout approaches.

While our scRNA-seq analysis delineated the cell-type-specific features of pyrimidine metabolism in the JEV-infected mouse brain, it did not spatially resolve metabolic differences across distinct brain regions. Existing evidence indicates that pyrimidine metabolism exhibits regional heterogeneity, with the hippocampus showing comparably stronger *de novo* synthesis,^24^ which may underpin its high functional and neurogenic activity.^25^^,^^26^ JEV displays marked neurotropism for developing neurons and neural progenitor cells,^27^, ^28^, ^29^ and the hippocampus is a key target for infection and injury.^30^^,^^31^ Based on our finding that JEV depends on host *de novo* pyrimidine biosynthesis for its replication, we hypothesize that this metabolic dependency may contribute to JEV's regional tropism for the hippocampus. This hypothesis remains to be experimentally tested.

The increased demand for metabolic precursors imposed by viral replication appears to be accompanied by coordinated changes in glutamine utilization. We observed reduced expression of key enzymes involved in intracellular glutamine metabolism and dynamic changes in glutamine transporter expression, suggesting a shift toward increased reliance on extracellular glutamine. Notably, the glutamine transporter SLC1A5 was transiently upregulated early in infection but declined at later time points, a pattern consistent with a previous study on Japanese encephalitis.^32^ Pharmacological inhibition of SLC1A5 did not significantly impair viral replication at early stages and even enhanced replication at later stages, suggesting that blocking a single transporter may not be sufficient to limit glutamine availability. Our scRNA seq analysis revealed upregulation of alternative glutamine transporters, including SLC38A1 and SLC38A2, in highly infected neurons. Similar compensatory upregulation of these transporters has been reported in SLC1A5-deficient cancer cells under conditions of glutamine limitation, supporting the possibility of transporter redundancy.^33^ However, this interpretation is based on transcriptional evidence and does not establish a causal role for these transporters. Future loss of function studies will be required to validate the proposed compensatory mechanism. Collectively, these findings highlight the metabolic flexibility of host cells during JEV infection and suggest that glutamine acquisition may be supported by multiple transport systems.

While our study elucidates the dependence of JEV on the *de novo* pyrimidine biosynthesis pathway in neurons, the contribution of the salvage synthesis pathway to viral replication remains to be fully characterized. Notably, our uridine rescue experiment demonstrates that exogenous uridine completely restores JEV replication under BQR treatment, confirming that the salvage pathway remains functional during infection and can be flexibly engaged when exogenous substrates are abundantly supplied. This finding suggests that the availability of salvage pathway substrates, such as uridine, may be a key limiting factor. Under physiological conditions, the local concentration of circulating nucleosides in the CNS microenvironment may be insufficient to meet the dramatically elevated nucleotide demand imposed by viral replication, thereby rendering the *de novo* pathway indispensable. Whether JEV also actively modulates salvage pathway activity or alters nucleoside transporter expression to facilitate substrate acquisition represents an interesting question for future investigation.

Several limitations should be considered in this study. Our conclusion of metabolic reprogramming relies on transcriptomic, enzyme protein expression, and pharmacological evidence. Direct liquid chromatography–tandem mass spectrometry quantification of pyrimidine nucleotide pools remains to be performed in future studies to definitively confirm the increased flux through the *de novo* synthesis pathway. Due to the lack of target validation, it remains uncertain whether V9302 at the employed concentrations is entirely specific for SLC1A5 in our study, and aminooxyacetate is a general inhibitor of pyridoxal phosphate-dependent enzymes without absolute specificity for GOT1/2. Genetic deletion approaches will be important to further validate the specific roles of these targets. Furthermore, the sample size (*n* = 3 Mock, *n* = 5 JEV) limits the statistical power for analyses of rare infiltrating immune cell subsets. Notably, these cell populations are largely absent in the normal mouse brain, further restricting comparative analyses between Mock and infected conditions. The metabolic features of these populations should therefore be viewed as descriptive and hypothesis‑generating. Additionally, the current evidence is limited to *in vitro* and *ex vivo* systems. Further *in vivo* experiments in JEV‑infected animal models are warranted to evaluate the translational potential of targeting pyrimidine metabolism in the CNS.

## 5 Conclusions

This study described the cell-type-specific transcriptional landscape of pyrimidine metabolism in both normal and JEV-infected mouse brains at single-cell resolution. JEV infection enhanced *de novo* pyrimidine biosynthesis pathway in neurons, which is essential for viral replication. Furthermore, JEV critically depends on exogenous glutamine uptake and GOT-mediated endogenous aspartate production. Collectively, these findings define cell-type-resolved metabolic dependencies that highlight potential targets for future antiviral strategies.

## CRediT authorship contribution statement

**Jun Gu:** Writing – review & editing, Writing – original draft, Visualization, Software, Resources, Project administration, Methodology, Investigation, Formal analysis, Data curation, Conceptualization. **Chencheng Fan:** Visualization, Validation, Software, Investigation, Formal analysis. **Shengxian Xiang:** Validation, Investigation. **Youhui Si:** Supervision, Resources. **Bibo Zhu:** Supervision, Resources. **Shengbo Cao:** Writing – original draft, Visualization, Supervision, Resources, Funding acquisition, Conceptualization. **Jing Ye:** Writing – review & editing, Writing – original draft, Visualization, Validation, Supervision, Resources, Funding acquisition, Conceptualization.

## Informed consent

Informed consent is not required, as this study did not involve any human subjects.

## Organ donation

Not applicable.

## Ethical statement

Not applicable.

## Data availability statement

The authors declare that all data supporting the findings of this study are available within the article and its supplementary materials.

## Animal treatment

The single-cell RNA sequencing data analyzed in this study were derived from a prior study that was approved by the Institutional Animal Care and Use Committee of Huazhong Agricultural University, as detailed in Yang et al. (2024).^13^ Primary mouse brain mixed neuron/glia cultures were prepared from neonatal mice under a protocol approved by the Committee on the Ethics of Animal Experiments of Huazhong Agricultural University (Permit No. HZAUMO-2025-0117).

## Generative AI

Generative AI and AI-assisted technologies were used exclusively for language editing and improving the readability of the manuscript. No AI tools were used for data analysis, interpretation of results, or the generation of scientific content.

## Funding

This work was supported by National Key Research and Development Program of China (2023YFC2306501 and 2022YFD1801500), National Natural Science Foundation of China (32372993 and U25A20715), Fundamental Research Funds for the Central Universities (2662025DKPY004), and Technology Innovation Base (Platform) Plan of Hubei Province (2025CSA092).

## Declaration of competing interest

Authors Shengbo Cao and Jing Ye are members of the Editorial Board of this journal. To ensure the integrity of the editorial process, they were not involved in the peer review or editorial decision-making for this manuscript. The other authors declare that they have no competing interests.

## Appendix Supplementary materials

Image, application 1
