# Supplementary material for: Japanese encephalitis virus reprograms host pyrimidine metabolism to facilitate viral replication
Source: Infect Med (Beijing). 2026 Jul 31;5(3):100274. doi: 10.1016/j.imj.2026.100274 (PMC13571495; doi:10.1016/j.imj.2026.100274)
Supplement: Supplementary file 1 [file mmc1.docx]

Supplementary Fig. S1 CMPK2 overexpression suppresses JEV replication.

N2a cells transfected with Flag-CMPK2 or empty vector were infected with JEV (MOI = 5) at 24 h post-transfection, and viral protein level was quantified by western blots at 18 and 24 hpi.

*Abbreviations*: CMPK2, Cytidine/uridine monophosphate kinase 2; JEV, Japanese encephalitis virus; MOI, multiplicity of infection; N2a, Neuro-2a.

Supplementary Fig. S2 Validation of key metabolic dependencies in primary mouse brain mixed neuron/glia cultures.

(A) Representative immunofluorescence image of primary mixed neuron/glia cultures showing neuronal (MAP2) and glial (GFAP) markers.

(B) Time-course analysis of DHODH protein expression in primary cultures infected with JEV (MOI = 1). β-actin served as a loading control.

(C) BQR treatment suppresses JEV replication in primary cultures, rescued by uridine. Cells were infected with JEV (MOI = 1) and treated with BQR (10 μM) with or without 200 μM uridine. Viral RNA and titer were quantified by qRT-PCR and plaque assay at 18 hpi.

(D) Expression of GOT1 and GOT2 proteins in JEV-infected primary cultures. Cells were harvested at 36 hpi and analyzed by immunoblotting.

(E) AOA treatment reduces JEV replication in primary cultures. Primary cells were infected (MOI = 1), treated with AOA (200 μM) at 1.5 hpi, and viral RNA and titer were measured at 24 hpi.

Data are presented as mean ± standard error of the mean from 3 independent biological replicates (n = 3). Significance was determined using two-tailed unpaired Student’s t-test (E) and one-way ANOVA (C) (***p* < 0.01, ****p* < 0.001; *****p* < 0.0001).

*Abbreviations*: ANOVA, analysis of variance; AOA, aminooxyacetic acid; BQR, brequinar; DHODH, dihydroorotate dehydrogenase; JEV, Japanese encephalitis virus; MOI, multiplicity of infection; PNG, primary mixed neuron/glia cultures; qRT-PCR, quantitative reverse transcription polymerase chain reaction.

Table S1. List of key pyrimidine metabolism and transporter genes.

| **Pathway** | **Gene** | **Protein** | **EC Number** | **Catalytic activity/Function** |
| --- | --- | --- | --- | --- |
| *De novo* pyrimidine synthesis | *Cad* | Carbamoyl-phosphate synthetase 2, Aspartate transcarbamylase, and Dihydroorotase | 6.3.5.5 | hydrogencarbonate + L-glutamine + 2 ATP + H2O = carbamoyl phosphate + L-glutamate + 2 ADP + phosphate + 2 H+ |
|  |  |  | 2.1.3.2 | carbamoyl phosphate + L-aspartate = N-carbamoyl-L-aspartate + phosphate + H+ |
|  |  |  | 3.5.2.3 | (S)-dihydroorotate + H2O = N-carbamoyl-L-aspartate + H+ |
| *De novo* pyrimidine synthesis | *Dhodh* | Dihydroorotate dehydrogenase | 1.3.5.2 | (S)-dihydroorotate + a quinone = orotate + a quinol |
| *De novo* pyrimidine synthesis | *Umps* | Uridine 5'-monophosphate synthase | 2.4.2.10 | orotidine 5'-phosphate + diphosphate = orotate + 5-phospho-alpha-D-ribose 1-diphosphate |
|  |  |  | 4.1.1.23 | orotidine 5'-phosphate + H+ = UMP + CO2 |
| *De novo* pyrimidine synthesis | *Ctps1* | CTP synthase 1 | 6.3.4.2 | UTP + L-glutamine + ATP + H2O = CTP + L-glutamate + ADP + phosphate + 2 H+ |
| *De novo* pyrimidine synthesis | *Ctps2* | CTP synthase 2 | 6.3.4.2 | UTP + L-glutamine + ATP + H2O = CTP + L-glutamate + ADP + phosphate + 2 H+ |
| *De novo* pyrimidine synthesis | *Cmpk* | UMP-CMP kinase | 2.7.4.14 | (d)CMP + ATP = (d)CDP + ADP, (d)UMP + ATP = (d)UDP + ADP |
| *De novo* pyrimidine synthesis | *Cmpk2* | UMP-CMP kinase2 | 2.7.4.14 | (d)CMP + ATP = (d)CDP + ADP, (d)UMP + ATP = (d)UDP + ADP |
| Pyrimidine salvage and catabolism | *Uck1* | Uridine-cytidine kinase 1 | 2.7.1.48 | uridine + ATP = UMP + ADP + H+, cytidine + ATP = CMP + ADP + H+ |
| Pyrimidine salvage and catabolism | *Uck2* | Uridine-cytidine kinase 2 | 2.7.1.48 | uridine + ATP = UMP + ADP + H+, cytidine + ATP = CMP + ADP + H+ |
| Pyrimidine salvage and catabolism | *Uckl1* | Uridine-cytidine kinase-like 1 | 2.7.1.48 | uridine + ATP = UMP + ADP + H+, cytidine + ATP = CMP + ADP + H+ |
| Pyrimidine salvage and catabolism | *Dck* | Deoxycytidine kinase | 2.7.1.74 | 2'-deoxycytidine (or deoxyadenosine, deoxyguanosine) + a ribonucleoside 5'-triphosphate = dCMP (or dAMP, dGMP) + a ribonucleoside 5'-diphosphate + H+ |
| Pyrimidine salvage and catabolism | *Dpyd* | Dihydropyrimidine dehydrogenase [NADP(+)] | 1.3.1.2 | 5,6-dihydrouracil(or dihydrothymine) + NADP+ = uracil(or thymine) + NADPH + H+ |
| Pyrimidine salvage and catabolism | *Dpys* | Dihydropyrimidinase | 3.5.2.2 | 5,6-dihydrouracil + H2O = 3-(carbamoylamino)propanoate + H+ |
| Pyrimidine salvage and catabolism | *Upb1* | Beta-ureidopropionase | 3.5.1.6 | 3-(carbamoylamino)propanoate(or 3-(carbamoylamino)-2-methylpropanoate) + H2O + 2 H+ = beta-alanine(or (R)-3-amino-2-methylpropanoate) + NH4+ + CO2 |
| Pyrimidine salvage and catabolism | *Upp1* | Uridine phosphorylase 1 | 2.4.2.3 | (2'-deoxy)uridine+ phosphate = (2'-deoxy-)alpha-D-ribose 1-phosphate + uracil |
| Pyrimidine salvage and catabolism | *Upp2* | Uridine phosphorylase 2 | 2.4.2.3 | (2'-deoxy)uridine+ phosphate = (2'-deoxy-)alpha-D-ribose 1-phosphate + uracil |
| Pyrimidine salvage and catabolism | *Cda* | Cytidine deaminase | 3.5.4.5 | (2'-deoxy)cytidine + H2O + H+ = (2'-deoxy)uridine + NH4+ |
| dNTP synthesis and quality control | *Rrm1* | Ribonucleoside-diphosphate reductase large subunit | 1.17.4.1 | a 2'-deoxyribonucleoside 5'-diphosphate + [thioredoxin]-disulfide + H2O = a ribonucleoside 5'-diphosphate + [thioredoxin]-dithiol |
| dNTP synthesis and quality control | *Rrm2* | Ribonucleoside-diphosphate reductase subunit M2 | 1.17.4.1 | a 2'-deoxyribonucleoside 5'-diphosphate + [thioredoxin]-disulfide + H2O = a ribonucleoside 5'-diphosphate + [thioredoxin]-dithiol |
| dNTP synthesis and quality control | *Rrm2b* | Ribonucleoside-diphosphate reductase subunit M2 B | 1.17.4.1 | a 2'-deoxyribonucleoside 5'-diphosphate + [thioredoxin]-disulfide + H2O = a ribonucleoside 5'-diphosphate + [thioredoxin]-dithiol |
| dNTP synthesis and quality control | *Dut* | Deoxyuridine 5'-triphosphate nucleotidohydrolase | 3.6.1.23 | dUTP + H2O = dUMP + diphosphate + H+ |
| dNTP synthesis and quality control | *Dctpp1* | dCTP pyrophosphatase 1 | 3.6.1.12 | dCTP + H2O = dCMP + diphosphate + H+ |
| dNTP synthesis and quality control | *Dctd* | Deoxycytidylate deaminase | 3.5.4.12 | dCMP + H2O + H+ = dUMP + NH4+ |
| dNTP synthesis and quality control | *Dtymk* | Thymidylate kinase | 2.7.4.9 | dTMP + ATP = dTDP + ADP |
| Nucleoside phosphate interconversion | *Nme1* | Nucleoside diphosphate kinase | 2.7.4.6 | a 2'-deoxyribonucleoside 5'-diphosphate + ATP = a 2'-deoxyribonucleoside 5'-triphosphate + ADP |
| Nucleoside phosphate interconversion | *Nme2* | Nucleoside diphosphate kinase | 2.7.4.6 | a 2'-deoxyribonucleoside 5'-diphosphate + ATP = a 2'-deoxyribonucleoside 5'-triphosphate + ADP |
| Nucleoside phosphate interconversion | *Nme3* | Nucleoside diphosphate kinase C | 2.7.4.6 | a 2'-deoxyribonucleoside 5'-diphosphate + ATP = a 2'-deoxyribonucleoside 5'-triphosphate + ADP |
| Nucleoside phosphate interconversion | *Nme4* | Nucleoside diphosphate kinase D, mitochondrial | 2.7.4.6 | a 2'-deoxyribonucleoside 5'-diphosphate + ATP = a 2'-deoxyribonucleoside 5'-triphosphate + ADP |
| Nucleoside phosphate interconversion | *Nme6* | Nucleoside diphosphate kinase 6, mitochondrial | 2.7.4.6 | a 2'-deoxyribonucleoside 5'-diphosphate + ATP = a 2'-deoxyribonucleoside 5'-triphosphate + ADP |
| Nucleoside phosphate interconversion | *Nme7* | Nucleoside diphosphate kinase 7 | 2.7.11.1 | L-seryl-[protein] + ATP = O-phospho-L-seryl-[protein] + ADP + H+ |
| Nucleotide dephosphorylation | *Nt5c* | 5'(3')-deoxyribonucleotidase, cytosolic type | 3.1.3.5 | a ribonucleoside 5'-phosphate + H2O = a ribonucleoside + phosphate |
| Nucleotide dephosphorylation | *Nt5c1a* | Cytosolic 5'-nucleotidase 1A | 3.1.3.5 | a ribonucleoside 5'-phosphate + H2O = a ribonucleoside + phosphate |
| Nucleotide dephosphorylation | *Nt5c1b* | Cytosolic 5'-nucleotidase 1B | 3.1.3.5 | a ribonucleoside 5'-phosphate + H2O = a ribonucleoside + phosphate |
| Nucleotide dephosphorylation | *Nt5c2* | Cytosolic purine 5'-nucleotidase | 3.1.3.5 | a ribonucleoside 5'-phosphate + H2O = a ribonucleoside + phosphate |
| Nucleotide dephosphorylation | *Nt5c3b* | 7-methylguanosine phosphate-specific 5'-nucleotidase | 3.1.3.5 | a ribonucleoside 5'-phosphate + H2O = a ribonucleoside + phosphate |
|  |  |  | 3.1.3.91 | N7-methyl-GMP(or CMP) + H2O = N7-methylguanosine(cytidine) + phosphate |
| Nucleotide dephosphorylation | *Nt5e* | 5'-nucleotidase | 3.1.3.5 | a ribonucleoside 5'-phosphate + H2O = a ribonucleoside + phosphate |
| Nucleotide dephosphorylation | *Nt5m* | 5'(3')-deoxyribonucleotidase, mitochondrial | 3.1.3.5 | a ribonucleoside 5'-phosphate + H2O = a ribonucleoside + phosphate |
| Pyrimidine nucleoside transport | *Slc29a1* | Equilibrative nucleoside transporter 1 | NA | Transport of nucleosides and nucleobases |
| Pyrimidine nucleoside transport | *Slc29a2* | Equilibrative nucleoside transporter 2 | NA | Transport of nucleosides and nucleobases |
| Pyrimidine nucleoside transport | *Slc28a1* | Sodium/nucleoside cotransporter 1 | NA | Sodium/pyrimidine nucleoside symporter that uses the Na+ gradient to drive cellular uptake of nucleosides and nucleobases. |
| Pyrimidine nucleoside transport | *Slc28a2* | Sodium/nucleoside cotransporter 2 | NA | Sodium/pyrimidine nucleoside symporter that uses the Na+ gradient to drive cellular uptake of nucleosides and nucleobases. |
| Pyrimidine nucleoside transport | *Slc28a3* | Solute carrier family 28 member 3 | NA | Sodium/pyrimidine nucleoside symporter that uses the Na+ gradient to drive cellular uptake of nucleosides and nucleobases. |
| Pyrimidine nucleoside transport | *Slc25a33* | Solute carrier family 25 member 33 | NA | Mitochondrial transporter that imports/exports pyrimidine nucleotides into and from mitochondria. |
| Pyrimidine nucleoside transport | *Slc25a36* | Solute carrier family 25 member 36 | NA | Mitochondrial transporter that imports/exports pyrimidine nucleotides into and from mitochondria. |

The information was obtained from Universal Protein Resource (UniProt) and Kyoto Encyclopedia of Genes and Genomes (KEGG) databases.

Table S2. Differential expression statistics for pyrimidine metabolism genes discussed in Section 3.2.

| **Cell** | **Gene** | **Comparison** | ***p*_val** | **avg_log2FC** | **pct.1** | **pct.2** | ***p*_val_adj** |
| --- | --- | --- | --- | --- | --- | --- | --- |
| **Astrocyte** | ***Gls*** | JEV-P_vs_Mock | 0.3248 | -0.0252 | 0.1670 | 0.2630 | 1.0000 |
| **Astrocyte** | ***Glul*** | JEV-P_vs_Mock | 0.2490 | -0.2723 | 0.9000 | 0.9120 | 1.0000 |
| **Astrocyte** | ***Got1*** | JEV-P_vs_Mock | 0.5375 | 0.0062 | 0.3000 | 0.2250 | 1.0000 |
| **Astrocyte** | ***Got2*** | JEV-P_vs_Mock | 0.0072 | 1.4721 | 0.3330 | 0.1510 | 1.0000 |
| **EC** | ***Cmpk2*** | JEV-P_vs_Mock | <0.0001 | 3.6449 | 0.8100 | 0.1210 | <0.0001 |
| **EC** | ***Ctps*** | JEV-P_vs_Mock | <0.0001 | 2.6365 | 0.4950 | 0.0670 | <0.0001 |
| **EC** | ***Gls*** | JEV-P_vs_Mock | <0.0001 | -1.0767 | 0.3390 | 0.3550 | 0.3685 |
| **EC** | ***Glul*** | JEV-P_vs_Mock | <0.0001 | -2.0567 | 0.6420 | 0.7880 | <0.0001 |
| **EC** | ***Got1*** | JEV-P_vs_Mock | <0.0001 | 0.0655 | 0.2970 | 0.1560 | <0.0001 |
| **EC** | ***Got2*** | JEV-P_vs_Mock | 0.5627 | -0.7745 | 0.2580 | 0.2140 | 1.0000 |
| **Microglia/MΦ** | ***Cmpk2*** | JEV-P_vs_Mock | <0.0001 | 5.6874 | 0.5160 | 0.0100 | <0.0001 |
| **Microglia/MΦ** | ***Gls*** | JEV-P_vs_Mock | <0.0001 | -1.6849 | 0.2460 | 0.3420 | <0.0001 |
| **Microglia/MΦ** | ***Glul*** | JEV-P_vs_Mock | <0.0001 | -2.7662 | 0.4520 | 0.8400 | <0.0001 |
| **Microglia/MΦ** | ***Got1*** | JEV-P_vs_Mock | <0.0001 | 0.8261 | 0.5220 | 0.1990 | <0.0001 |
| **Microglia/MΦ** | ***Got2*** | JEV-P_vs_Mock | <0.0001 | -0.2430 | 0.2480 | 0.1510 | <0.0001 |
| **Monocytes/MΦ** | ***Ctps*** | JEV-P_vs_JEV-N | 0.0561 | 2.0043 | 0.0280 | 0.0050 | 1.0000 |
| **Monocytes/MΦ** | ***Gls*** | JEV-P_vs_JEV-N | 0.0149 | -0.6169 | 0.1930 | 0.2490 | 1.0000 |
| **Monocytes/MΦ** | ***Glul*** | JEV-P_vs_JEV-N | 0.0001 | -0.8389 | 0.3220 | 0.4130 | 1.0000 |
| **Monocytes/MΦ** | ***Umps*** | JEV-P_vs_JEV-N | 0.0399 | 0.5386 | 0.1010 | 0.0530 | 1.0000 |
| **Mural** | ***Cmpk2*** | JEV-P_vs_Mock | <0.0001 | 5.9614 | 0.6110 | 0.0100 | <0.0001 |
| **Mural** | ***Ctps*** | JEV-P_vs_Mock | <0.0001 | 2.1953 | 0.3020 | 0.0410 | <0.0001 |
| **Mural** | ***Gls*** | JEV-P_vs_Mock | <0.0001 | -1.2854 | 0.2050 | 0.2530 | 0.4097 |
| **Mural** | ***Glul*** | JEV-P_vs_Mock | 0.2049 | -0.3111 | 0.3970 | 0.2980 | 1.0000 |
| **Mural** | ***Got1*** | JEV-P_vs_Mock | <0.0001 | 0.8070 | 0.4250 | 0.1570 | <0.0001 |
| **Mural** | ***Got2*** | JEV-P_vs_Mock | 0.1597 | -0.4676 | 0.2410 | 0.1840 | 1.0000 |
| **Neuron** | ***Cmpk2*** | JEV-P_vs_Mock | <0.0001 | 3.9937 | 0.3480 | 0.0270 | <0.0001 |
| **Neuron** | ***Gls*** | JEV-P_vs_Mock | <0.0001 | -1.2512 | 0.4330 | 0.5120 | <0.0001 |
| **Neuron** | ***Glul*** | JEV-P_vs_Mock | <0.0001 | -1.0983 | 0.3340 | 0.3480 | <0.0001 |
| **Neuron** | ***Got1*** | JEV-P_vs_Mock | <0.0001 | 0.1294 | 0.4750 | 0.3110 | <0.0001 |
| **Neuron** | ***Got2*** | JEV-P_vs_Mock | <0.0001 | -0.2007 | 0.3570 | 0.2430 | <0.0001 |
| **NK** | ***Cad*** | JEV-P_vs_JEV-N | 0.5874 | -0.3191 | 0.0150 | 0.0000 | 1.0000 |
| **NK** | ***Ctps*** | JEV-P_vs_JEV-N | 0.3253 | 0.6772 | 0.0460 | 0.0000 | 1.0000 |
| **NK** | ***Gls*** | JEV-P_vs_JEV-N | 0.2288 | 0.6280 | 0.2300 | 0.1000 | 1.0000 |
| **NK** | ***Glul*** | JEV-P_vs_JEV-N | 0.1256 | -1.0672 | 0.3200 | 0.4000 | 1.0000 |
| **NK** | ***Got1*** | JEV-P_vs_JEV-N | 0.0299 | -1.4923 | 0.3030 | 0.4500 | 1.0000 |
| **NK** | ***Got2*** | JEV-P_vs_JEV-N | 0.3626 | 0.1142 | 0.1990 | 0.1000 | 1.0000 |
| **NK** | ***Slc1a5*** | JEV-P_vs_JEV-N | 0.0787 | 1.2504 | 0.2250 | 0.0500 | 1.0000 |
| **NK** | ***Tyms*** | JEV-P_vs_JEV-N | 0.3686 | 0.8806 | 0.0390 | 0.0000 | 1.0000 |
| **NK** | ***Uck1*** | JEV-P_vs_JEV-N | 0.1634 | 1.8335 | 0.0890 | 0.0000 | 1.0000 |
| **NK** | ***Uck2*** | JEV-P_vs_JEV-N | 0.2362 | 1.3829 | 0.0660 | 0.0000 | 1.0000 |
| **OLG** | ***Cmpk2*** | JEV-P_vs_Mock | <0.0001 | 8.1319 | 0.7610 | 0.0080 | <0.0001 |
| **OLG** | ***Gls*** | JEV-P_vs_Mock | <0.0001 | -0.9593 | 0.2140 | 0.2950 | <0.0001 |
| **OLG** | ***Glul*** | JEV-P_vs_Mock | <0.0001 | -0.7638 | 0.8620 | 0.8880 | <0.0001 |
| **OLG** | ***Got1*** | JEV-P_vs_Mock | <0.0001 | -0.1281 | 0.3140 | 0.2370 | 0.5409 |
| **OLG** | ***Got2*** | JEV-P_vs_Mock | 0.0144 | -0.3266 | 0.3540 | 0.2810 | 1.0000 |
| **T** | ***Gls*** | JEV-P_vs_JEV-N | 0.0128 | -0.6114 | 0.3450 | 0.3930 | 1.0000 |
| **T** | ***Glul*** | JEV-P_vs_JEV-N | 0.0035 | -0.7210 | 0.3110 | 0.3750 | 1.0000 |
| **T** | ***Slc1a5*** | JEV-P_vs_JEV-N | <0.0001 | 1.6750 | 0.2920 | 0.0830 | 0.0010 |
| **T** | ***Uck2*** | JEV-P_vs_JEV-N | 0.0057 | 0.9991 | 0.1040 | 0.0360 | 1.0000 |
| **T** | ***Umps*** | JEV-P_vs_JEV-N | 0.0140 | 0.6420 | 0.0940 | 0.0360 | 1.0000 |
| **T** | ***Got1*** | JEV-P_vs_JEV-N | 0.0016 | 0.6041 | 0.5140 | 0.3810 | 1.0000 |
| **T** | ***Got2*** | JEV-P_vs_JEV-N | 0.7839 | -0.3559 | 0.2690 | 0.2500 | 1.0000 |
| **OPC** | ***Cmpk2*** | JEV-P_vs_Mock | <0.0001 | 4.6668 | 0.7120 | 0.0360 | <0.0001 |

This table provides statistical details for genes explicitly discussed in Section 3.2.
